# Climate Change and Health: A Study of the Attitudes of Future Science Teachers

**DOI:** 10.3390/ijerph22010007

**Published:** 2024-12-24

**Authors:** María Rocío Pérez-Mesa, Yair Alexander Porras-Contreras, Rosa Nidia Tuay-Sigua

**Affiliations:** 1 Biology Department, National Pedagogical University, Bogota 110221, Colombia; 2 Chemistry Department, National Pedagogical University, Bogota 110221, Colombia; yporras@pedagogica.edu.co; 3 Physical Department, National Pedagogical University, Bogota 110221, Colombia; rtuay@pedagogica.edu.co

**Keywords:** climate change, health, attitudes, teachers in training, science education

## Abstract

Living beings as open systems depend on climate and weather to survive. However, changes in the Earth’s climatology, which have become more frequent since the industrial period, have affected different territories of the planet, limiting access to ecosystem services and causing imbalances in health and well-being. The first purpose of this study is to conduct a literature review on academic production regarding climate change and its impact on health, in the context of education, using international academic production condensed in the Web of Science (WOS) database over the last 10 years as a reference. The second purpose focuses on identifying the environmental attitudes of science teachers in initial training regarding aspects related to climate change. The study results show three categories emerging from the literature review: Climate Change and Health, Nature and Risks, and Environment and Energy. For the analysis of environmental attitudes, a survey was conducted with 51 pre-service teachers, consisting of 59 items distributed in five categories: (a) environment, (b) climate change, (c) health, (d) education, and (e) lifestyle. Although the results reveal a positive attitude towards all analyzed categories, it is important to advance effective mitigation and adaptation strategies from the teacher training processes themselves.

## 1. Introduction

In a period of history associated with profound transformations in the way reality is conceived, new categories emerge in the relationship between humans and other beings of nature, caused by the establishment of a crisis scenario and a panorama of inequalities and inequities, which invite the construction of new bases for environmental sustainability, recognizing its potential in the processes of citizen formation [1]. Given the evidence of global deterioration, associated with anthropogenic causes that have been escalating since the industrial era [2], it is important to promote studies that account for the relationship between human activities and global environmental transformations, such as climate change [3], biodiversity loss [4], difficult access to resources, and excessive waste generation. In this sense, the analysis of variables associated with social and cultural practices that promote transformations in the Earth’s system [5] constitutes proof of the influence of human actions on the global geological system, a fact evidenced by transformations in Earth’s dynamics, such as the “Great Acceleration” [6], a concept that includes the rapid increase in human population, the rise in CO_2_ concentration in the atmosphere, and the increasing temperature figures that have made the last 10 years the warmest in the planet’s recent history [7]. Climate change implies health risks due to increasingly hostile climate variability, requiring more urgent measures to ensure the quantity and quality of food and water, reduce air pollution, control vector distribution, and decrease disease transmission [8].

In recent years, greater importance has been given to biodiversity and its impact on human well-being and health, especially considering the close link between biological diversity and health from a systemic perspective, coupled with a complex perspective that includes the interrelationships between various forms of life, with humans embedded in this web. In this sense, biodiversity provides direct and indirect benefits to society, especially through climate regulation, water provision and regulation, air quality, food security, disaster prevention and mitigation, and recreation, which contribute to well-being and benefits for physical, mental, emotional, and social health [9].

The first purpose of this research is to conduct a literature review on education, climate change, and its impact on health, using international academic production condensed in the Web of Science (WOS) database, an academic platform known for its scientific impact, the diversity of journals it includes, and its broad thematic coverage [10,11]. A second objective of this study focuses on identifying the ideas expressed by pre-service science teachers about climate change and its impacts on health, highlighting the commitment to environmental sustainability and decision-making. In line with these objectives, this article aims to answer the following research questions:

- What are the most frequently mentioned research topics by authors on education regarding climate change and the influence of this issue on health?
- What attitudes do future science teachers express towards climate change and its impact on health?

## 2. Theoretical Framework

The theoretical framework that guides this research is presented below, focusing the discussion on topics such as climate change, lifestyle, environmental attitudes and environmental attitudes of science teachers.

### 2.1. Climate Change

Climate change is defined as “any change in climate over time, whether due to natural variability or as a result of human activity” [12]. Criticisms of this definition, which coincide with some objections to the concept of the Anthropocene, focus on the empowerment of the environmental field by groups and organizations that express a position of global dominance, close to technical governance. In the face of this panorama of instrumentalization of the environmental field, it is necessary to reaffirm an emancipatory principle in which people cannot be subordinated to the fluctuations of the economy; on the contrary, they demand a protagonism that allows them to build a possible present and future, far from the impositions of the prevailing development model.

The thesis on the impact of climate-related factors on the health and well-being of the population is gaining strength. In this regard, the Pan American Health Organization of the Region of the Americas establishes a close relationship between a highly complex phenomenon such as climate change and human health. In this regard, it warns about the disruption of global physical, biological, and ecological systems by climate change, which causes impacts on the health and well-being of communities. This phenomenon could affect well-being conditions through various pathways associated with extreme weather events, such as intense heat waves or increased flooding, air pollution, food insecurity, changes in the distribution of vector-borne diseases, and disaster risk. The World Health Organization [13] states that climate change is a reality that affects people’s lives and also points out that the health risks of climate change are not equitable between and within regions and communities, which is why it warns that this phenomenon can contribute to increasing social inequalities and can also lead to the deterioration of ecosystems, constituting a spiral in which socioeconomic, environmental, and health conditions can be highly compromised.

The challenges that shape a scenario of inequities and inequalities, from which environmental identity emerges, allow a process of empowerment of social groups around the recognition of beliefs, conceptions, and social representations that people build regarding aspects that affect their lives [14]. From this perspective, environmental education focused on mitigating or adapting to climate change becomes a political and pedagogical purpose, strengthening an environmental education process that has as its central axis the initial recognition of the social representations circulating in communities [15], with the aim of configuring a field of action in which the work of teachers who take on the challenge of addressing climate change from an innovative and investigative perspective is made visible.

### 2.2. The Lifestyle

Lifestyle comprises the set of daily actions and habits that people adopt to satisfy their individual and collective needs. An unsustainable lifestyle has a direct impact on health and environmental conditions, due to the irrational management of natural resources, the production of greenhouse gasses in daily activities, pollution, hyper-consumption of energy, excessive production of solid waste, the destruction of natural territories, and the decrease or loss of biodiversity [16]. The lifestyle of modern society seeks to satisfy certain needs through the acquisition of products provided by the industry, and which result in a wide offer, but which in some cases have had incalculable impacts on the environment and the health of ecosystems. As an example, it has been estimated that the presence of microplastics in the ocean could reach 150 million tons, and up to 8 million tons of plastics can enter the sea annually [17]. Much of this material corresponds to single-use plastics (cups, cutlery, trays, plates, straws, bags), which have spread to all continents and have been incorporated into the daily activities of people and communities, and only in recent years have they begun to be regulated by laws to contribute to their reduction in the environment. In recent years, another problem that has arisen is the presence of microplastics that include a wide range of polymers and chemical additives [18], which can be ingested by a variety of aquatic organisms, with the potential to cause them harm and the possibility of entering the human diet [19].

Another component corresponds to the transportation sector, which favors the production of goods and services by allowing the movement of workers, students, and the community in general and, in turn, facilitates the transfer of goods to markets for consumer acquisition. The transportation sector model for cities has been based on the use and increase in private vehicles, freight transport, and public transport. Most of these means of transport, based on the use of fossil fuels, have generated negative impacts on the environment, which are related to the impact on air quality, its impact on global climate change, public health, and quality of life in cities, since it is estimated that this sector is the one that most affects air pollution in Colombian cities [20]. In recent decades, scientific research has studied the effects of different pollutants on human health, and particularly on respiratory, neurological, and cardiac diseases [21,22].

### 2.3. Environmental Attitudes

Attitudes are considered a polysemic construct that can be confused with beliefs, values, dispositions, and personal norms. According to a consensus of specialists, an attitude refers to a predisposition to action mentally linked to a concrete or abstract object [23], which, from a three-dimensional model, consists of three components: affective (manifested as a feeling of liking or disliking); cognitive (beliefs or opinions); and behavioral (behaviors or actions) [24,25,26,27]. In the environmental field, the affective dimension involves environmental awareness [28], initially considered as a set of psychological factors related to individuals’ predisposition to participate in pro-environmental activities. Consequently, environmental attitudes are defined as “concern for the environment or concern for environmental issues” [29].

Environmental attitudes, when related to environmental values, can be defined as “a psychological tendency expressed by evaluating the natural environment with some degree of favorability or unfavorability” [30,31]. It is important to recognize that environmental concern is a part of environmental attitude, which is defined as “an evaluation or attitude towards facts, one’s own behavior, or the behavior of others with consequences for the environment” [32]. More recently, some studies have highlighted the historical and contextual perspective of environmental attitudes, particularly the ideological polarization on socially relevant issues, which becomes an important variable to promote citizens’ decision-making in a period of environmental crisis [33].

In recent decades, a series of investigations have been conducted aimed at studying environmental attitudes and other relevant constructs, especially considering the fundamental contributions to science education, environmental education, and environmental psychology, among other factors. It is worth noting that, given the current challenges related to socio-environmental issues, climate change, and its impacts on health, it is a priority to place these issues on international and local agendas for society and education, to shed light on the gaps, ruptures, or distances in the relationships between humans and nature, which cross through a complex vision of the environment, linking different dimensions at social, economic, political, cultural, educational, and ethical levels. Several investigations and authors agree on the need to understand attitudes and, through pedagogical and didactic proposals, contribute to change [34], to achieve environmental sustainability that recognizes the complexity of the environment, socio-environmental issues, climate change, and its possible impacts on human health, so that educational processes are oriented towards improving conditions for health, well-being, and the protection of various forms of life.

### 2.4. Environmental Attitudes of Science Teachers

Some studies related to the environmental attitudes of science teachers in the face of climate change show a growing interest in promoting a didactic and pedagogical treatment of global environmental problems from the personal sphere, which is sometimes unfavorable due to increasing feelings of guilt and helplessness, which translate into ineffective learning activities with little commitment on the part of students [35,36]. In general terms, teachers’ attitudes are very close to transnational public opinion on climate change [37], particularly the gap between saying and doing, recurrent in most countries, as they show concern for the current situation of the planet but with a low willingness to make sacrifices and thus reverse global risks [38].

In a qualitative study where the opinions of chemistry teachers on the teaching of climate change were evaluated, it was revealed that there is a certain consensus on the importance of learning central aspects of climate change, without being clear about the place that this topic should occupy in the curriculum [39]. The inclusion of the environmental dimension in school curricula is also no guarantee that the issue of climate change will be addressed in a systematic way. In fact, the interest of some teachers in promoting learning about climate change is directly related to social representations that consider attitudes and knowledge of climate change mitigation behaviors as central axes of environmental training, in addition to consolidating themselves as a means to empower young citizens with the construction of a possible world [40].

A study focused on identifying the social representations of climate change among future biology teachers reveals that their environmental attitudes are linked to cognitive, affective, and action aspects. Therefore, most participants attribute the responsibility for the planet’s deterioration to anthropogenic activities [15]. When investigating the central core of the social representation of climate change among pre-service teachers through prototypical and categorical analysis, six words with higher frequency and lower evocation rank were found: pollution, human activities, unconsciousness, consumerism, temperature, and carbon dioxide. This confirms the interest of future teachers in understanding the lack of awareness and inaction of citizens towards transforming a hyper-consumerist lifestyle away from the principles of environmental sustainability. In the peripheral zone, words directly related to the central core of climate change are found: climate, deforestation, melting, crisis, alteration, logging, and imbalance. These terms reflect a problematic view of environmental reality, highlighting the environmental imbalances that determine the risk landscape, which validates the perspective of late modernity, marking a separation between humans and nature.

## 3. Materials and Methods

This research is of a mixed type, allowing a broad understanding of the research phenomenon based on contemporary problems such as climate change and its impact on health, located in a relevant field such as the training of science teachers. In this sense, it integrates both qualitative and quantitative aspects of the method. Mixed designs refer to the type of study, in which the researcher combines research techniques, methods, and approaches, quantitative or qualitative, in a single investigation [41]. These designs provide varied, even divergent, points of view of the phenomenon or approach under study and allow a more complete and comprehensive approach to the phenomenon studied, since they are based on the pragmatic paradigm [42].

**Figure 1:**
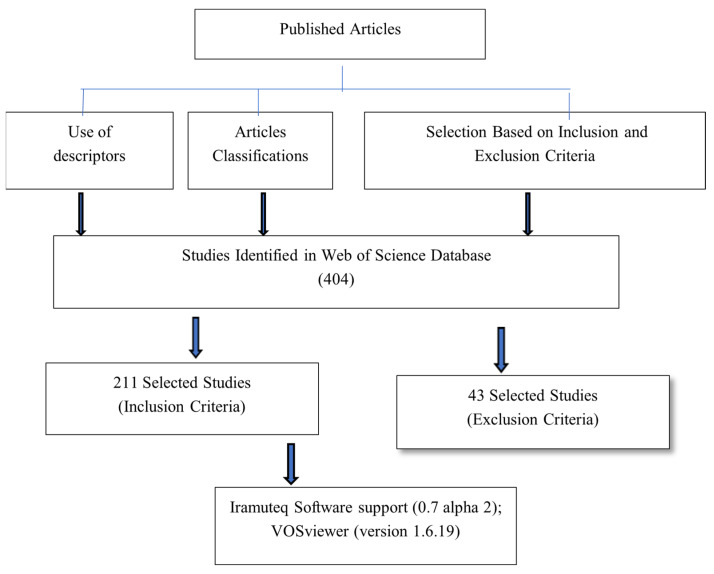
Document review flowchart. Source: authors.

The first phase includes a review that adopts the principles of qualitative research, particularly with a methodological approach to the state of the art, which consists of “inventorying and systematizing production in a certain area of knowledge. But it is also one of the qualitative modalities of “research of research” that seeks to systematize the work carried out within a given area, a review of sources and documents is carried out, to comply with a descriptive level” [43]. This phase contemplates the identification of trends and key problems through the study with bibliometric parameters, which allow for the identification of lines of research that have been configured around climate change and health (Figure 1). The systematic review associated with the topic in question allows a joint vision to be provided of what scientific evidence says about that problem [44]. In this sense, qualitative Systematic Reviews (SR) in which statistical methods are not applied focus on providing qualitative assessments of said results, and in this type of review statistical analysis techniques can be applied, which turn an SR into a meta-analysis [45]. The above implies the use of search strategies and inclusion criteria as a series of exclusion filters [46]. For this first phase, a search process was carried out for articles published in one of the databases with the highest impact factor and visibility, at an international level: Web of Science. This site was searched with the Boolean code detailed below. A search process was carried out for articles published in the Web of Science database, from 2010 to 2024, entering the following Boolean code: climate change AND education AND health AND teacher AND school. The reason for choosing this time interval is to focus on relating climate and public policy events in Colombia that began in 2010 and determined the increase in environmental awareness and decision-making regarding climate change mitigation or adaptation. In fact, 2010 was the first warmest year on record in the 21st century since systematic measurements began in 1880, as the combined global land and ocean surface temperature was 0.62 °C above the 20th century average [47]. On the other hand, in Colombia, the National Plan for Adaptation to Climate Change was defined in 2010 as one of the strategies aimed at reducing vulnerability to this problem and preparing the country to adapt to climate change.

The inclusion criteria were contemplated in a dichotomous and qualitative way, classifying works as “accepted” or “rejected” depending on whether they met the following criteria:

1. Academic works or scientific articles that are in the field of study or other related fields;
2. Published during the last 15 years (2010 to 2024);
3. Published in scientific journals with indexation;
4. Studies with direct or indirect reference to climate change, health, and the environment.

A bibliographic matrix was constructed using Microsoft Office Excel to integrate those articles that met the inclusion criteria. These were recorded with the following data, author(s), title of the article, year of publication, journal, keywords, abstract, language in which it was published, participants, and research scenarios, and 404 articles were found. Regarding the process of excluding publications, it was carried out based on two aspects. The first corresponds to those articles that did not make direct reference to the representational field of education, climate change, and the impacts on health, which led to 211 articles being obtained. A second exclusion criterion corresponds to those articles whose research was carried out in areas other than the educational field. From this review, 43 articles with 218 words in three clusters were obtained. Likewise, keywords that included climate change, health, education, and science teacher training were taken into account. At the international level, there is a growing number of publications that explore the field of attitudes about climate change and its impacts on the general public; however, studies that specifically investigate the attitudes of science teachers in training and in service are scarce [48,49]. In the same way, teachers’ attitudes and social representations in the face of environmental problems such as climate change determine aspects of their professional teaching performance [16], which demonstrates a correlation between teachers’ perceptions, environmental knowledge, attitudes, and teaching practices [50,51]. By way of synthesis, studies focused on analyzing teachers’ perceptions and attitudes towards environmental problems, including climate change, reveal a relatively limited understanding of environmental citizenship, collective responsibility, and alternatives to face global risks. These studies demonstrate teachers’ attitudes that are limited to the local scale, the individual dimension, and the private sphere, affecting teaching practices and teachers’ professional identity. It is clear that attitudes towards climate change can be improved during teacher education, through programs that include the development of professional competences based on scientific knowledge, analysis of the context, and the empowerment of teachers.

**Figure 2:**
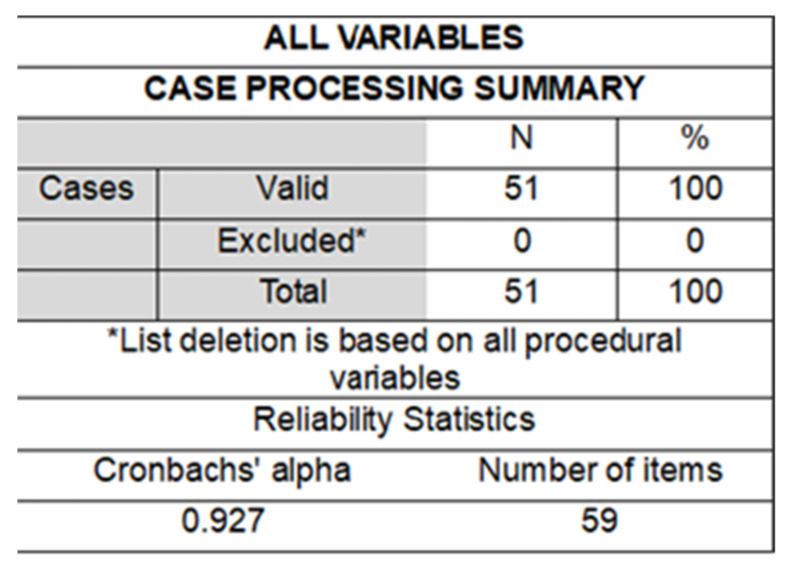
Cronbach’s alpha result. Source: SPSS software results.

The second phase focuses on descriptive quantitative methods, using the non-probabilistic convenience sampling technique, which allows for the selection of accessible cases that show the proximity of the subjects to the researcher [52]. This study was developed at the National Pedagogical University, located in Bogotá (Colombia). The sample consisted of 51 pre-service science teachers (24 biology, 15 chemistry, and 12 physics), of whom 67% were women and 33% were men, aged between 18 and 26 years. In Colombia, teacher training is carried out in Faculties of Education, where the title of Licensed is awarded, with which graduates can work as high school teachers in their specific field of study. The instrument used is a closed questionnaire that contains an Attitude Scale with the following categories: (a) environment, (b) climate change, (c) health, (d) education, and (e) lifestyle. This instrument is composed of 59 items. The scale was answered in a 5-point format (strongly agree to strongly disagree). It is worth noting that, within the ethical criteria of the research, participants were previously informed of the study’s objective and its educational scope, without using proper names and maintaining anonymity. Therefore, they were provided with all the information and expressed their interest in the study, in addition to accepting their participation with informed consent. To estimate the reliability of the instrument, Cronbach’s alpha was calculated using the Statistical Package for the Social Sciences (SPSS version 25), with a value of 0.927 (Figure 2), indicating high consistency and measurement precision.

In recent decades, a series of investigations have been carried out aimed at studying environmental attitudes and other relevant constructs, especially when considering the fundamental contributions to science education, environmental education, and environmental psychology [53,54,55]. It should be noted that, given the current challenges related to socio-environmental problems, climate change and its impacts on health, it is a priority to place these issues on international and local agendas for society and education, in order to shed light on the gaps and ruptures or distancing in the relationships between human beings and nature, which cross a complex vision of the environment, in which different dimensions intersect at the epistemological, ontological, social, economic, political, cultural, educational, and ethical levels. Several studies and authors agree on pointing out the need to know people’s attitudes to contributing to change through pedagogical and didactic proposals [38] and to achieving an environmental sustainability that is reflected in better conditions for the various forms of life.

For the treatment of the data, the IRAMUTEQ program (version 0.7 alpha 2) was used in order to proceed with the multidimensional analysis of texts, in this case the summaries of the articles obtained in the review of the WOS database. This free software allows you to encode the summaries of the texts, and to later generate graphics of textual statistics, word clouds, analyses of specificities, and the Descending Hierarchical Classification (CDJ). Consequently, the visualization of bibliometric networks using the VOSviewer software (version 1.6.19) allowed us to obtain a lexicographic analysis of thematic groups (clusters) related to climate change, health, and teachers’ ideas on these topics. This second phase of the study aims to identify the ideas expressed by a group of pre-service science teachers on climate change and its impact on health, recognizing the attitudes they manifest towards environmental issues and aspects of critical thinking such as decision-making and analytical skills.

## 4. Results

### 4.1. Research Topics in the Field of Climate Change and Health

The bibliometric analysis allowed for the recognition of keywords representing the central themes of the publications, which constitute the research topics described in the 43 scientific articles studied from the WOS database. To understand and visualize the structure and relationships between the examined documents, it is necessary to establish research nodes, from which bibliometric networks were constructed. Among the most popular software for visualizing these bibliometric networks is VOSviewer, which allows for the visualization of citation patterns, co-citations, bibliographic coupling, keyword co-occurrence, and co-authorship networks. These bibliometric networks can be visualized from three approaches: distance-based, graph-based, and timeline-based [56].

**Figure 3:**
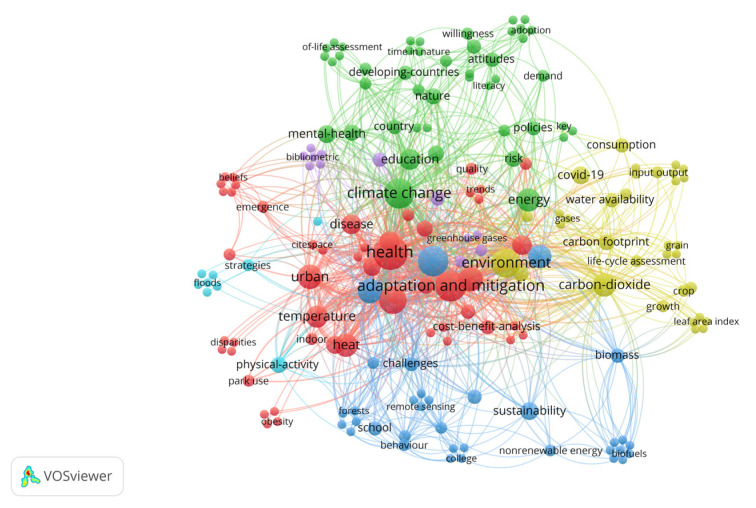
Visualization of research topics education, climate change, and health. Source: authors, based on VOSviewer.

The keyword co-occurrence analysis developed through VOSviewer software allowed for the identification of terms that appear most frequently in publications on education regarding climate change and health, as well as the words most strongly associated with the field’s topics. Figure 3 presents the network visualization map of research topics in education regarding climate change and its impact on health. Three distinct groups are represented in different colors: climate change and health in red, nature and risks in green, and environment and energy in blue.

**Figure 4:**
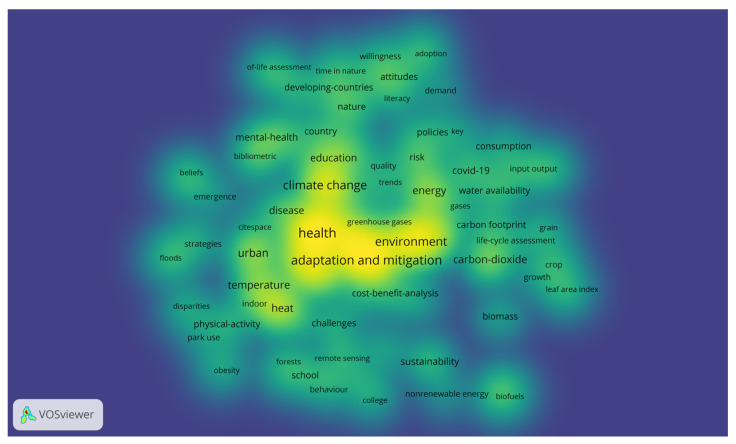
Density visualization of climate change, education, and health. Source: authors, based on VOSviewer.

Figure 4 shows the frequency or co-occurrence of terms in the text corpus on climate change education and health. This density map or heat map from VOSviewer graphically represents the relationship between the most frequently used words and their grouping or relationship in three clusters. The graph highlights the relationship between climate change and health, and the adaptation and mitigation processes to deal with specific environmental problems, such as air pollution, increased CO_2_, vulnerability, and mortality.

**Figure 5:**
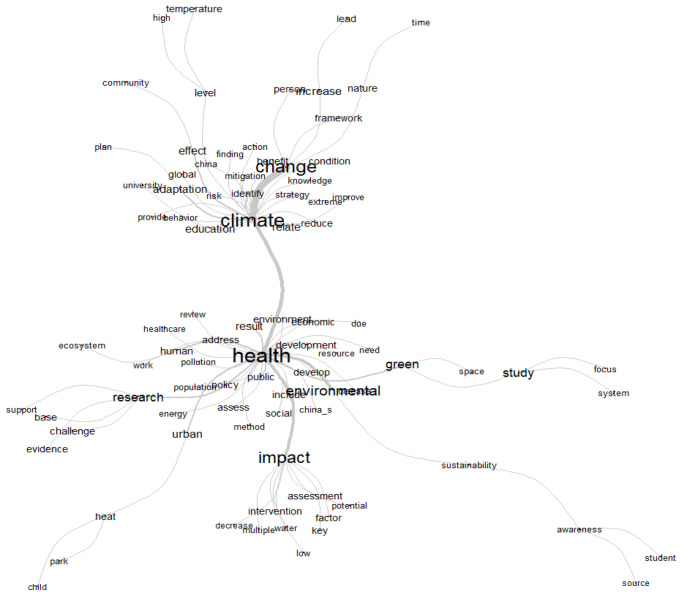
Similarity chart: education against climate change and health. Source: authors, based on IRAMUTEQ.

The IRAMUTEQ software (Interface de R pour les Analyses Multidimensionnelles de Textes et de Questionnaires) [57] allowed us to carry out a lexicometric study associated with the group of publications on education regarding climate change and health, which analyzed text segments characteristic of the representational field constituted by the reviewed articles. The general corpus was composed of 43 texts (a summary of each article), which was divided into 278 text segments. The textual statistical analysis showed that 9897 occurrences (words, forms, or vocabulary) arose, of which 1218 appeared only once in the entire corpus analyzed. Figure 5 shows the IRAMUTEQ similarity graph, which highlights strong relationships, expressed by the thickest connection lines between the terms climate change, health, impacts, and environment. Other words that reveal relevant relationships in the representational field are increase, study, research, urban, and development.

### 4.2. Attitudes of Pre-Service Teachers on Climate Change and Health

The attitudinal trend of pre-service teachers towards the environment is satisfactory, as evidenced by the percentage of positive responses in the eight items analyzed, which reached 81%. The highest-rated items were as follows: “1. Caring for the environment is everyone’s responsibility” (98%), and “34. Solid waste thrown into rivers and streams is a pollution factor that can increase the risk of flooding during rainy seasons” (91%). Among the items with a score of 62% was “2. Caring for the environment is mainly the responsibility of the government and environmental entities”, which contrasts with the highest-rated items, considering that the environment is a matter for government entities, alluding to an assistentialist perspective with a relative commitment from communities. In this sense, personal commitment may come into tension with attributing responsibility to government entities. Likewise, it is highlighted that for item 2, “Humans can transform the environment without restrictions to meet their needs”, the responses were concentrated in the ‘Strongly Disagree’ option, followed by the ‘Disagree’ option, while for item 11, “Humans must meet their needs without affecting the environment for future generations”, 80% of the responses were concentrated in the ‘Strongly Agree’ and ‘Agree’ options, indicating that these attitudes correspond more to an eco-centric vision. Although the answers of pre-service teachers are similar to those from other studies [15,38,39,40], particularly in aspects related to environmental responsibility attributable to governments and corporations, there is an environmental awareness of the need to change the anthropocentric paradigm on which the global environmental crisis is fostered.

#### 4.2.1. Education Category

The attitudinal trend of pre-service science teachers is satisfactory for the education category, with the percentage of positive responses in the seven items analyzed reaching 85%. The highly rated items included the following: “27. The creation of school or community gardens allows for the recognition of the importance of organic farming and the improvement of healthy eating habits”, and “40. It is necessary to separate solid waste (organic and inorganic) from home”, with most responses concentrated in the ‘Strongly Agree’ option, both reaching 94%. Another item that received a high percentage was item 29, “The development of environmental projects in school settings promotes environmental education and community participation with sustainable practices”, with 92% of responses concentrated in the ‘Strongly Agree’ and ‘Agree’ options. In this sense, participants recognize the roles of home, family, and school as places and institutions that can contribute to the formation of attitudes in favor of the environment and health through the implementation of strategies such as school gardens to promote sustainable practices and proper solid waste management. On the other hand, item 40, “The only way to implement energy-saving strategies is by increasing the service bill rate”, showed a higher number of responses in the ‘Disagree’ option, followed by the ‘Strongly Disagree’ option, reaching 76%, indicating that the attitude towards saving should not be conditioned by external agents, especially if it involves conditioning savings with an increase in service payment.

#### 4.2.2. Climate Change Category

Regarding the category related to climate change, it was found that the attitudinal tendency of the initial science teachers was satisfactory, showing a percentage of positive responses in the 10 items analyzed, which reached 84%. The items with the highest ratings were as follows: “56. Climate change worsens food insecurity due to heat waves or heavy rains” (94%); “51. Climate change can cause health problems for people” (92%), and “48. Climate change causes major impacts on agricultural production and food security” (91%). In this regard, it is important to highlight that most of the responses were for the option ‘Totally agree’, followed by the option ‘Agree’, especially when noting the complexity of this phenomenon and its impact on human health due to extreme weather events (heat waves, floods) and the repercussions for food security that can impact and generate greater gaps in populations. Regarding item 57, “The increase in greenhouse gas emissions can increase the risk of more floods, illnesses and deaths” (87%), it was found that the majority of responses were located in the options ‘Totally Agree’ and ‘Agree’, associating the presence and increase in these gasses with climate change, air pollution, and the risk they can represent for life, health, and the environment in general, results that are related to the statements of world organizations [11].

It should be noted that for item 55, “Despite floods and droughts, populations have adapted to remain in the same place without major impacts”, a response of (63%) was obtained, with the majority of responses present in the option ‘Neither in Agreement nor in Disagreement’, followed by the option ‘In Disagreement’. These responses may be related to the intense meteorological phenomena that have occurred in the country and to the fact that, due to sometimes late or limited response mechanisms, populations return to these places despite the potential risks that they may represent for their health and well-being.

#### 4.2.3. Health Category

The attitudinal tendency of initial science teachers for the health category was found to be satisfactory, with a percentage of positive responses to the 13 items analyzed, which reached 89%. The best-valued items were as follows: item 7, “Air pollution can cause an increase in respiratory diseases” (95%), item 8, “The increase in greenhouse gas emissions represents a risk to life on the planet” (94%), and item 3, “The loss of biodiversity can affect public health and put the permanence of life at risk” (91%). These percentages of responses provided by the participants were concentrated mainly in the options ‘Totally Agree’ and ‘Agree’. That is to say, they present attitudes that show the degree of agreement they can have regarding the relationship between respiratory diseases and air pollution, and they also go beyond the vision of human health and the local context to express their agreement regarding the risk of increased greenhouse gasses and the diversity of life on the planet or the loss of biodiversity and its impact on health and the continuity of life, which raises attitudes related to an eco-centric vision. Item 46, “The loss of soil fertility and desertification can affect the quality of the environment, food production and human health” (89%), also presented the highest number of responses for the options ‘Totally Agree’ and ‘Agree’. This aspect shows an openness beyond physical and mental health and includes a complex vision of the relationships between human beings, the social context, and the environment. For its part, item 33, “Rainwater can be used for human consumption without affecting health” (65%), obtained the lowest percentage compared to the other items, presenting a distribution of responses in the five options, reaching the highest concentration for the options ‘Neither Agree Nor Disagree’ and ‘Disagree’, an aspect that allows us to reflect on the vision of access to and consumption of drinking water that contrasts with the use of rainwater without any treatment and the possible effects on human health.

#### 4.2.4. Lifestyle Category

Regarding the lifestyle category, it was found that the attitudinal trend of pre-service science teachers remains satisfactory, showing a percentage of positive responses in the eight items analyzed, reaching 73%. The highest-rated items were as follows: item 21, “Changing habits in the use of all types of plastics at home reduces pollution (92%)”, item 32, “I enjoy visiting parks with large green areas where I can engage in leisure activities and improve my health” (91%), and item 38, “Single-use plastics (cups, cutlery, trays, plates, straws, bags) should be removed from circulation because they pollute the environment” (89%). These items allow us to appreciate the degree of agreement with attitudes related to the willingness to change habits regarding the use of plastics at home and similarly show correspondence with the importance of eradicating single-use plastics due to their negative impact on the environment. They also positively value being able to enjoy visiting parks or green areas as part of actions and habits for their health and well-being.

In this category, some items received a low percentage of responses from the participants, as detailed below: item 24, “Cleaning and hygiene products are chosen at home for their low cost, without considering their components” (45%); item 23, “Public mass transportation is insufficient for the number of users in the city, so the presence of private cars and motorcycles is necessary”. This means that, although they are young people training to be science teachers, the choice of cleaning products is based on household economy rather than the components and effects they may have on health and the environment. Regarding the type of transportation, there are certain tensions regarding the increase in population in cities, the availability of public transportation, and the increase in private vehicles, which are reflected in the responses provided by the participants, choosing the option ‘Neither Agree nor Disagree’. It is likely that this is still seen as an issue for other instances to address.

## 5. Discussion

Health has historically been defined as “a state of complete physical, mental, and social well-being, and not merely the absence of disease or infirmity” [58], a concept that clearly advocates for an integral balance in various aspects of life. This broad definition does not escape criticism, as it becomes the center of discussions when an absolutist view of well-being is pointed out, in addition to a static perspective that does not consider health as a dynamic and historical concept that depends on different periods of crisis. In order to articulate the educational implications of studying climate change and its repercussions on health, some reflections on the most representative results of the bibliometric review are developed below, along with the analysis of the attitudes evidenced by pre-service science teachers, with the aim of fostering the construction of elements for decision-making in the formation of environmentally responsible citizens.

Physical, mental, emotional, and social health is influenced by the way people develop their own personal potential, interact with the social environment, and respond to environmental changes. Physical, mental, and social health establishes a complex vision of the relationships between humans, the social context, and the environment, which at different scales allows for the dimensioning of the web of relationships between humans, themselves, others, and nature [59]. In this sense, individual and collective well-being may be at risk due to those impacts that alter ecosystems, particularly anthropogenic activities, which put the continuity of life at risk, along with the deterioration of water quality, air quality, and the loss of fertile soils, as well as hyper consumption and inadequate management of organic and inorganic waste, which translates into potential risks and threats to human health and the fragility of the permanence of various forms of life [60].

The links between human health, the environment, and biodiversity are woven from a complex vision that seeks to overcome a fragmented view of the world, especially considering humans as a biological species with cultural and social developments that have led to great transformations throughout their time on the planet. Firstly, in the history of humanity, food as an essential basis for health and the sustenance of life introduced one of the greatest changes through domestication and the invention of agriculture, with a series of practices that transformed ecosystems [61], which over the centuries expanded in different regions of the planet, potentially increasing risks at the interface between humans and the environment. Rapid population growth and concentration in urban environments exert greater pressure on ecosystems both within the urban perimeter and nearby ecosystems [62], due to the high demand for water services, electricity, and other energy sources, requiring the intervention of reserve areas for the supply of these services, such as hydroelectric plants. Likewise, urban expansion, the introduction of invasive species, and habitat destruction, along with climate change, can create an environment that favors the emergence of pathogens and represents a significant threat to biodiversity and health in general.

Air pollution from greenhouse gas emissions from fixed and mobile sources [63], and the increase in particulate matter from forest fires or sands from the Sahara in an interconnected planet, among other factors, have caused cities like Bogotá [64] to issue alerts due to poor air quality, and the government has established measures such as restricting outdoor recreational and sports activities and restricting vehicle mobility, among other measures, to counteract the impact on potential diseases in children, the elderly, and pregnant women, and to maintain the integrity of ecosystems.

### 5.1. On Research Topics in the Field of Education, Climate Change, and Health

**Figure 6:**
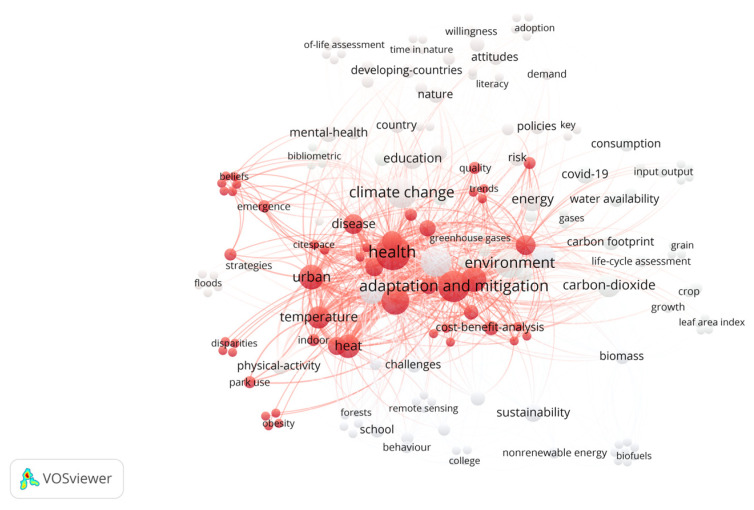
Cluster on climate change and health. Source: authors, based on VOSviewer.

The main threats to health from climate change include the danger of high temperatures and extreme storms, as well as less obvious aspects related to the survival, distribution, and behavior of mosquitoes, ticks, and rodents that become vectors of diseases [65]. Taking these ideas into account, the authors propose to consider the specific exposure pathways that can cause diseases in humans. Exposure pathways constitute a complex concept that allows us to understand the routes by which climate change affects health, without ignoring that this is a multi-level problem whose threats can accumulate over time, leading communities to consolidate self-organization processes for effective decision-making that involves adaptation and mitigation. According to the results obtained by the VOSviewer program in relation to the words with the highest frequency and greatest representational strength of the 43 articles analyzed, three clusters are presented that group together related terms: climate change and health (87 items), education and risks (72 items), and environment and energy (52 items). In the first cluster, climate change and health, the words with the highest frequency and strength appear, which include climate change, health, adaptation, and mitigation (Figure 6).

The importance of these terms confirms the weight given in the literature to adaptation, a process of designing, implementing, monitoring, and evaluating strategies, policies, and measures aimed at reducing the impacts of climate change [66]. The references in the literature to the increases in temperature, extreme heat, and air pollution confirm their association with infectious and respiratory diseases, cardiovascular and neurological pathologies [67], and heat stress, allergies, and food insecurity.

**Figure 7:**
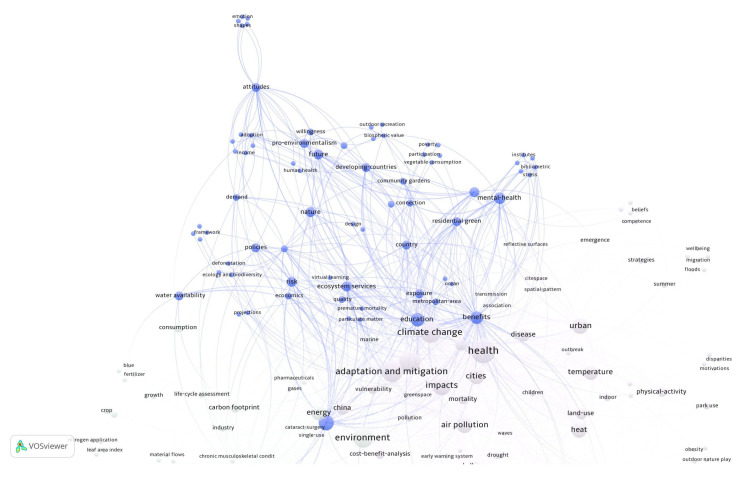
Cluster on education and risks. Source: authors, based on VOSviewer.

The second cluster, education and risks, includes words such as nature, education, policies, pro-environmental behaviors, ecosystem services, and economy, among others (Figure 7).

**Figure 8:**
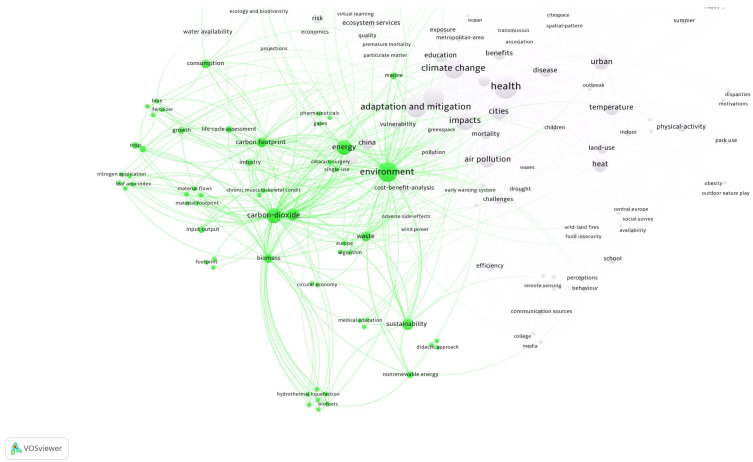
Cluster on environment and energy. Source: authors, based on VOSviewer.

The recent interest in climate change education is due, according to some authors [68], to the expansion of funding and leadership of educational programs that address climate change, the growing awareness of unusual weather patterns, and concern about the likelihood of global environmental, social, and economic changes. For pre-service science teachers, this period of uncertainty brings with it new challenges around climate change education, particularly the idea that it is difficult to talk about atmospheric and climate stability with students and citizens in general. However, a starting point is to investigate people’s ideas, perceptions, conceptions, and social representations about climate change. Among the results obtained in a recent study on social representations of climate change in pre-service science teachers [13], the reference to anthropic activities that threaten the ecological balance of the planet and the growing concern of future teachers about the lack of awareness of a lifestyle far removed from environmental sustainability stand out. The third cluster environment and energy, groups together the terms sustainability, carbon dioxide, carbon footprint, waste, consumption, and biomass, among others (Figure 8).

The environment is a complex construct mediated by cultural aspects and symbolic representations that play an important role in the interpretation of daily life or in the development of scientific knowledge [69]. The environment is a system that involves the natural, the social, and the cultural; hence, factors that threaten the ecological balance, such as the increase in the carbon footprint, unsustainability, and sociocultural inequalities, directly affect the health and well-being of living beings. In this sense, sustainability is recognized as a process that guarantees equity over time, thanks to the recognition of the coevolution between nature and culture, within a biocentric view of reality.

**Figure 9:**
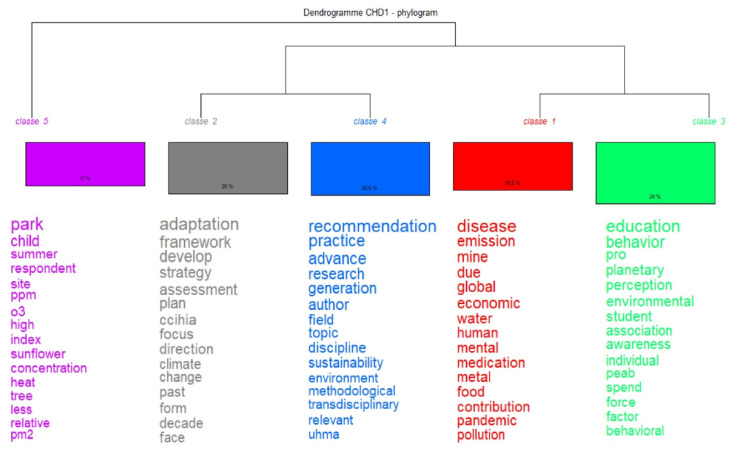
Dendrogram with the Descending Hierarchical Classification (DHC). Source: authors, based on IRAMUTEQ.

On the other hand, the content evaluated through the IRAMUTEQ software allowed the development of the Descending Hierarchical Classification (CHD), which is organized into five classes (Figure 9). Thus, the dendrogram generated from CHD presents the most recurrent terms and words in each of the classes that show the trends of the topics discussed.

**Figure 10:**
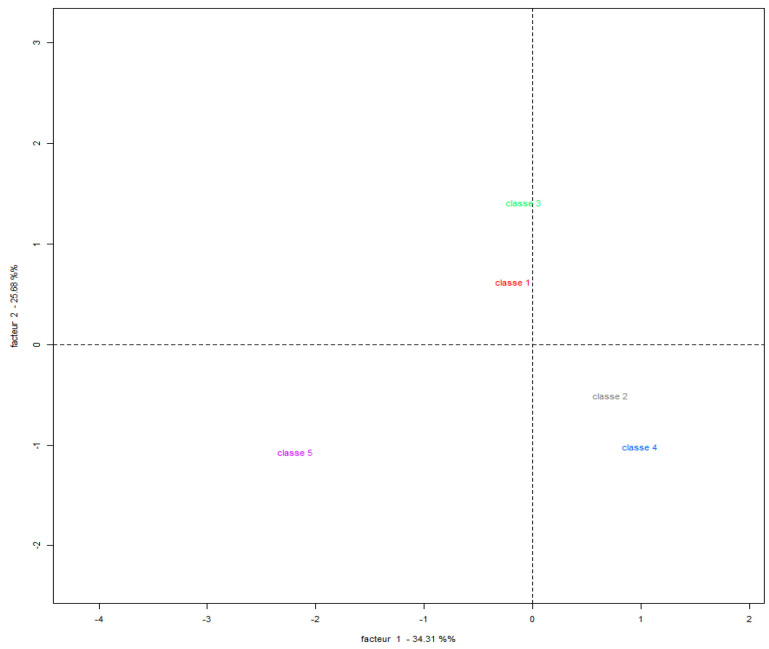
Cartesian plane Correspondence Factor Analysis (CFA). Source: authors, based on IRAMUTEQ.

Using Correspondence Factor Analysis (CFA), it was possible to create an association between words, considering the frequency and incidence of both the terms and the classes, so that they could be represented in the form of a Cartesian plane (Figure 10). It can be observed that the corpus had two large groups (subcorpus): Subcorpus 1 (classes 1, 2, 3, and 4) and Subcorpus 2 (class 5). From this interpretation, three categories of analysis result, since Subcorpus 1 represents two categories (classes 1–3 and classes 2–4) and Subcorpus 2 represents one category (class 5), based on the distance between the predominant words.

According to these results, the first category (classes 1 and 3) groups the terms that are associated with the words education and disease, highlighting the importance of education in the face of climate change from an adaptation perspective, which is associated with the eco-citizen proposal in which it is intended to raise awareness about the relationship between society and nature, promoting critical and creative thinking, in order to participate in decisions that affect the well-being of social groups [70].

### 5.2. On the Environmental Attitudes of Teachers in Initial Training

The study of attitudes towards the environment, climate change, health, lifestyle, and education in future science teachers provides valuable information that enriches the discussion on contemporary issues facing humanity. These issues can be explored in cognitive, affective, and behavioral terms, given their great relevance on an individual, citizen, and future educator level, under the paradigm of sustainability. These elements allow us to go beyond a recognition of socio-environmental problems, as the so-called environmental crisis encompasses a series of dispositional variables such as attitudes, beliefs, and values that can influence environmentally sustainable behaviors [71], which it is fundamental to review and address in educational processes.

The sustainability paradigm that emerged in the last decades of the 20th century allows for an approach from personal, social, and cultural spheres [72]. This research explores personal attitudes that include aspects of environmental psychology to understand their link with the environment, addressing questions about human–nature relationships, ways of valuing the environment, and attitudes towards climate change and its impact on health, as well as the commitment to lifestyles and their possible correspondence with sustainable practices. It is worth noting that there is a web of relationships between individuals, society, and culture regarding ways of conceiving the environment, developing lifestyles, and inhabiting the world, which can influence harmonious practices with nature concerning environmental sustainability.

Systemic and complex perspectives are part of the research work, providing insights into the responses provided by participants for each category. The complex approach allows for the interpretation of reality in terms of relationships and not just parts, which implies a distancing from simplifying principles that fragment reality into parts and limit its broad understanding [73]. In this way, the study of attitudes allows for a consideration of their relationships with individuals’ behaviors towards the environment, providing an approximation to understanding these relationships. Therefore, it is necessary to investigate them in the current environmental crisis, clarifying that this is only one aspect of this problem, which requires a multidimensional reading at social, cultural, economic, political, and ethical levels. Thus, attitudes allow an approach in their three components, cognitive, affective, and behavioral tendencies towards the natural environment, and in socially shared phenomena, symbols, or cultural practices.

Future science teachers show a favorable attitudinal trend towards the environment, as evidenced by items related to the physical environment and the valuation of socio-environmental problems, allowing them to identify their willingness to take actions in favor of conserving natural resources or showing their agreement with the sustainability paradigm, to consciously use resources that meet their needs without compromising availability or deteriorating the environment for future generations. Pre-service teachers recognize that humans benefit from natural resources in their role as consumers and identify problems associated with waste production from various anthropogenic activities, which can negatively impact the natural environment, allowing a protective behavior for environmental care to prevail [74].

From systemic and complex perspectives, it is important to highlight progress in overcoming fragmented worldviews, knowledge atomization, sustained exploitation logics, and dichotomies that separate humans from nature. Under this new framework, the phenomenon of climate change is assumed from a perspective that understands an interconnected planetary system, alert to the relationships established between humans and nature. This phenomenon also involves an ontological and epistemological view, posing a series of tensions and contradictions of the modernity project in economic development models and the use of fossil fuels in the industrial era, contrasting with new realities that have led to impacts of different orders and scales. Thus, in the responses provided by participants, the anthropogenic origin of climate change is recognized as a real problem, and their degree of agreement with the impacts it can have on human life, the permanence of various species, the progressive deterioration of some ecosystems, soil fertility loss, and the increase in extreme weather events is appreciated.

This panorama promotes an eco-centric perspective that reaffirms an interconnected vision of humans with biodiversity and the planet in general. Under this complexity approach, in line with the environment and the phenomenon of climate change accentuated since the industrial era, it is observed that students find a relationship between climate change and health. According to the responses provided in the health category, several items were rated with the highest percentages associated with their degree of agreement with the relationship between atmospheric pollution and the increase in respiratory diseases, biodiversity loss, and its impact on human health, or the relationship between vector-borne diseases (malaria, dengue) and their increase due to climate change. These results allow reflection on students’ understanding of the systemic representation that links humans as part of nature, obtaining goods and services, and involving an interconnected vision of the biosphere and the rhythms of anthropogenic activities, as well as the impact on health and well-being that extends to other living beings [75].

Regarding lifestyle, this category shows certain ambivalences towards the systemic and complex vision of the environment and their position as beings that are part of the web of life. In this sense, favorable attitudes are observed for some items regarding their willingness to enjoy certain spaces provided by the city, such as parks or green areas, or their willingness to change habits regarding the use of single-use plastics, recognizing the repercussions these have on the immediate environment’s deterioration and pollution from accumulation in soils and continental and oceanic water sources. However, regarding aspects such as the type of transportation used in the city and the increase in private vehicles, they show indifference to this problem, leaving aside an issue that continues to deepen in cities and is shown in various studies as the major emitters of greenhouse gasses in urban centers. Additionally, the possibility of choosing certain products is based more on economic benefits than environmental care, which requires special attention from society and education.

Finally, these results offer an important overview to consider the study of environmental attitudes from the educational field. In this sense, it is necessary to continue advancing in a systemic and complex vision of the environment that conceives the human being interrelated with the environment. In this regard, it is found that the knowledge, skills, and practices that are promoted from home and school can contribute to fostering pro-environmental behaviors that contribute to a paradigm of sustainability, in which the fragility of life in its various manifestations, the importance of adequate practices, and the willingness to promote changes in favor of the environment are recognized. In this sense, pedagogical practices can be directed to provide an education with a binding sense between the human being and the environment that contributes to providing conditions towards attitudes that allow conservation, the continuity of life, and the well-being of individuals and communities [76,77,78].

### 5.3. Limitations

While the findings provide significant insights into the attitudes of a group of pre-service science teachers towards an emerging issue like climate change, a limitation of the study is related to the selection bias of the sample, as it is not representative of the entire population of pre-service teachers in Bogotá.

It is important to note that future studies should address aspects related to the values underpinning each opinion of the pre-service teachers when addressing the topic of climate change. Consequently, this study allows for the recognition of a rarely addressed field of discussion in educational research, which involves the need to engage pre-service teachers in processes that enhance their environmental education, based on the opinions and attitudes they exhibit towards a controversial topic like climate change. It is necessary to consider for future studies the values underpinning each opinion of pre-service teachers when addressing the topic of climate change. Additionally, a crucial aspect of new research involves recognizing the professional teaching competencies that are deployed in teaching proposals arising from discussions within Faculties of Education. Consequently, this study allows for the recognition of a rarely addressed field of discussion in educational research, such as the need to engage pre-service teachers in processes that enhance their social representations, based on the opinions and attitudes they exhibit towards a controversial topic like climate change.

## 6. Conclusions

Climate change is becoming a threat to the survival of life on the planet. The study of climate variability and its consequences allows people to understand how these changes affect health and serve as a reference for informed decision-making in a mitigation or adaptation scenario, giving priority to research and innovation processes. Education regarding climate change becomes a priority in citizen training processes, particularly when addressing neuralgic aspects of the human condition such as uncertainty, risk, and change. An approach to bibliographic production around education, climate change, and the impacts on health reveals three priority categories in which research gaps are presented. The first of these, related to climate change and health, addresses those articles that show concern for the causes and consequences of environmental variability, particularly the repercussions of adverse conditions and the progression of pathologies over time. The education and risks category includes training processes within the framework of education regarding climate change, which allow us to face the environmental challenges that put communities on alert. It is important to recognize that in the environment and energy category, those articles that warn about planetary limits and the way to combat consumerism at different levels of interpretation of the global problem stand out.

The study of attitudes towards the environment, climate change, health, lifestyle, and education makes it possible to obtain new information from a generation of young people who are being trained as future science teachers, and their ways of conceiving the environment, climate change, and its impact on health can be key aspects to measure the complexity of these constructs, their assessments, and behaviors. The results obtained allow us to appreciate the satisfactory attitudinal tendencies for the majority of items that make up each of the categories and show an interrelated, systemic, and complex vision, which allows us to understand the possibilities of achieving greater involvement in correspondence with the paradigm of environmental sustainability, not through imposed and unconscious behaviors, but through a process of critical training from their role as citizens and future teachers in the face of the assessment of environmental complexity, the realities of sociocultural contexts, and the critical reading of current development models and lifestyles, which can contribute to educational reflection by making visible the tensions between anthropocentric, biocentric, and eco-centric perspectives, in a period that requires greater commitments towards the sustainability of life.

## Data Availability

All data generated or analyzed during this study are included in this published article.

## References

[1] Tuay-Sigua R.N., Pérez-Mesa M.R., Porras-Contreras Y.A. (2023). Teachers’ Ideas and Educational Experiences Regarding Urban Environmental Sustainability in Bogotá, Colombia. Sustainability.

[2] Steffen W., Grinevald J., Crutzen P., McNeill J. (2011). The Anthropocene: Conceptual and historical perspectives. Philos. Trans. R. Soc. A Math. Phys. Eng. Sci..

[3] Romanello M., McGushin A., Di Napoli C., Drummond P., Hughes N., Jamart L., Kennard H., Lampard P., Rodriguez B.S., Arnell N. (2021). The 2021 report of the Lancet countdown on health and climate change: Code red for a healthy future. Lancet.

[4] Yoshioka A., Miyazaki Y., Sekizaki Y., Suda S., Kadoya T., Washitani I. (2014). A “lost biodiversity” approach to revealing major anthropogenic threats to regional freshwater ecosystems. Ecol. Indic..

[5] Wals A., Weakland J., Corcoran P.B. (2017). Preparing for the Ecocene: Envisioning futures for environmental and sustainability education. Jpn. J. Environ. Educ..

[6] Head M.J., Steffen W., Fagerlind D., Waters C.N., Poirier C., Syvitski J., Zinke J. (2022). The Great Acceleration is real and provides a quantitative basis for the proposed Anthropocene Series/Epoch. Episodes.

[7] Tollefson J. (2023). Is it too late to keep global warming below 1.5 °C? The challenge in 7 charts. Nature.

[8] Tong S., Ebi K. (2019). Preventing and mitigating health risks of climate change. Environ. Res..

[9] Bermudez G.M.A., Perez-Mesa R., Ottogalli M.E. (2022). Biodiversity knowledge and conceptions in Latin American: Towards an integrative new perspective for education research and practice. Int. J. Educ. Math. Sci. Technol..

[10] Viehmann C., Fernández Cárdenas J.M., Reynaga Peña C.G. (2024). The Use of Socioscientific Issues in Science Lessons: A Scoping Review. Sustainability.

[11] Zhan Z., Shen W., Xu Z., Niu S., You G. (2022). A bibliometric analysis of the global landscape on STEM education (2004–2021): Towards global distribution, subject integration, and research trends. Asia Pac. J. Innov. Entrep..

[12] Intergovernmental Panel on Climate Change (2022). Climate Change 2022: Impacts, Adaptation, and Vulnerability. https://www.ipcc.ch/report/ar6/wg2/.

[13] World Health Organization (2024). Communicating on Climate Change and Health: Toolkit for Health Professionals.

[14] Martín-Páez T., Aguilera D., Perales-Palacios F.J., Vílchez-González J.M. (2019). What are we talking about when we talk about STEM education? A review of literature. Sci. Educ..

[15] Porras-Contreras Y.A., Pérez-Mesa M.R. (2019). Identidad ambiental: Múltiples perspectivas. Rev. Científica.

[16] Porras Y., Pérez R. (2022). Representaciones sociales del cambio climático en futuros profesores de Ciencias: Una mirada desde la perspectiva Freiriana. Tecné Epistem. Didaxis.

[17] Thacharodi A., Hassan S., Meenatchi R., Bhat M.A., Hussain N., Arockiaraj J., Ngo H.H., Sharma A., Nguyen H., Pugazhendhi A. (2024). Mitigating microplastic pollution: A critical review on the effects, remediation, and utilization strategies of microplastics. J. Environ. Manag..

[18] Blair Crawford C., Quinn B. (2016). Microplastic Pollutants.

[19] Wright S.L., Thompson R.C., Galloway T.S. (2013). The physical impacts of microplastics on marine organisms: A review. Environ. Pollut..

[20] Acosta G., Carrillo D., Caballero A. (2022). Microplásticos en agua y en organismos. Ciencia.

[21] Chowdhury R., Ramond A., O’Keeffe L.M., Shahzad S., Kunutsor S., Muka T., Gregson J., Willeit P., Warnakula S., Khan H. (2018). Environmental toxic metal contaminants and risk of cardiovascular disease: Systematic review and meta-analysis. BMJ.

[22] Fu Z., Ma Y., Yang C., Liu Q., Liang J., Weng Z., Li W., Zhou S., Chen X., Xu J. (2023). Association of air pollution exposure and increased coronary artery disease risk: The modifying effect of genetic susceptibility. Environ. Health.

[23] Gifford R., Sussman R., Clayton S.D. (2012). Environmental attitudes. The Oxford Handbook of Environmental and Conservation Psychology.

[24] Aragonés J., Amérigo M. (2000). Psicología Ambiental.

[25] Rosenberg M.J., Hovland C.I., McGuire W.J., Abelson R.P., Brehm J.W. (1960). Attitude Organization and Change: An Analysis of Consistency Among Attitude Components. (Yales Studies in Attitude and Communication).

[26] Ajzen I. (1971). Attitudinal vs. normative messages: An investigation of the differential effects of persuasive communications on behavior. Sociometry.

[27] Ajzen I., Fishbein M., Albarracin E., Johnson B.T., Zanna M.P. (2005). The influence of attitudes on behaviour. Handbook of Attitudes.

[28] Zelezny L., Schultz P. (2000). Psychology of Promoting Environmentalism: Promoting Environmentalism. J. Soc. Issues.

[29] Miller L.B., Rice R.E. (2024). (Mis)matched direct and moderating relationships among pro-environmental attitudes, environmental efficacy, and pro-environmental behaviors across and within 11 countries. PLoS ONE.

[30] Milfont T.L., Duckitt J. (2010). The environmental attitudes inventory: A valid and reliable measure to assess the structure of environmental attitudes. J. Environ. Psychol..

[31] Milfont T.L., Harre N., Sibley C.G., Duckitt J. (2012). The climate-change dilemma: Examining the association between parental status and political party support. J. Appl. Soc. Psychol..

[32] Fransson N., Gärling T. (1999). Environmental concern: Conceptual definitions, measurement methods, and research findings. J. Environ. Psychol..

[33] Birch S. (2019). Political polarization and environmental attitudes: A cross-national analysis. Environ. Politics.

[34] Páramo P. (2017). Reglas proambientales: Una alternativa para disminuir la brecha entre el decir-hacer en la educación ambiental. Suma Psicol..

[35] Aarnio-Linnanvuori E. (2019). How do teachers perceive environmental responsibility?. Environ. Educ. Res..

[36] Ahi B., Balci S., Alisinanoğlu F. (2017). Exploring Turkish preservice teachers’ mental models of the environ-ment: Are they related to gender and academic level?. J. Environ. Educ..

[37] Kim S.Y., Wolinsky-Nahmias Y. (2014). Cross-National Public Opinion on Climate Change: The Effects of Affluence and Vulnerability. Glob. Environ. Politics.

[38] Varela-Losada M., Vega-Marcote P., Lorenzo-Rial M., Pérez-Rodríguez U. (2021). The Challenge of Global Environmental Change: Attitudinal Trends in Teachers-In-Training. Sustainability.

[39] Feierabend T., Jokmin S., Eilks I. (2011). Chemistry teachers’ views on teaching ‘climate change’—An interview case study from research-oriented learning in teacher education. Chem. Educ. Res. Pract..

[40] Igboanugo B.I., Naiho O.I. (2024). Effectiveness of Inquiry-Based Chemistry Learning on Students’ Attitudes and Knowledge of Climate Change Mitigation Behaviors. J. Chem. Educ..

[41] Onwuegbuzie A.J., Johnson R.B. (2006). The Validity Issues in Mixed Research. Res. Sch..

[42] Mertens D. (2007). Transformative Paradigm: Mixed Methods and Social Justice. J. Mix. Methods Res..

[43] Garcés Á., Patiño C., Torres J.J. (2008). Juventud, investigación y saberes. Estado del arte de las Investigaciones sobre la Realidad Juvenil en Medellín 2004–2006.

[44] Booth A., Sutton A., Papaioannou D. (2016). Systematic Approaches to a Successful Literature Review.

[45] Montenegro-Rueda M., Fernández-Cerero J., Fernández-Batanero J.M., López-Meneses E. (2023). Impact of the Implementation of ChatGPT in Education: A Systematic Review. Computers.

[46] Page M.J., McKenzie J.E., Bossuyt P.M., Boutron I., Hoffmann T.C., Mulrow C.D., Shamseer L., Tetzlaff J.M., Akl E.A., Brennan S.E. (2021). The PRISMA 2020 statement: An updated guideline for reporting systematic reviews. J. Clin. Epidemiol..

[47] Barriopedro D., Fischer E.M., Luterbacher J., Trigo R.M., García-Herrera R. (2011). The Hot Summer of 2010: Redrawing the Temperature Record Map of Europe. Science.

[48] Liu S., Roehrig G.H., Bhattacharya D., Varma K. (2015). In-Service teachers’ attitudes, knowledge and classroom teaching of Global Climate Change. Sci. Educ..

[49] Georgiou Y., Hadjichambis A.C., Hadjichambi D. (2021). Teachers’ Perceptions on Environmental Citizenship: A Systematic Review of the Literature. Sustainability.

[50] Zachariou F., Tsami E., Chalkias C., Bersimis S. (2017). Teachers’ attitudes towards the environment and envi-ronmental education: An empirical study. Int. J. Environ. Sci. Educ..

[51] Petkou D., Andrea V., Anthrakopoulou K. (2021). The Impact of Training Environmental Educators: Environmen-tal Perceptions and Attitudes of Pre-Primary and Primary School Teachers in Greece. Educ. Sci..

[52] Otzen T., Manterola C. (2017). Técnicas de Muestreo sobre una Población a Estudio. Int. J. Morphol..

[53] Milfont T.L., Duckitt J. (2004). The structure of environmental attitudes: A firstand second-order confirmatory factor analysis. J. Environ. Psychol..

[54] Olsson D., Gericke N., Boeve-de Pauw J. (2022). The effectiveness of education for sustainable development revisited—A longitudinal study on secondary students’ action competence for sustainability. Environ. Educ. Res..

[55] Kirbiš A. (2023). Environmental Attitudes among Youth: How Much Do the Educational Characteristics of Parents and Young People Matter?. Sustainability.

[56] Van Eck N., Waltman L. (2014). Visualizing bibliometric networks. Measuring Scholarly Impact: Methods and Practice.

[57] Souza B.V., de Souza G., Ramos H.C. (2021). Sustainability Reporting in Higher Education Institutions: A systematic approach using VOSViewer and Iramuteq software. Int. J. Adv. Eng. Res. Sci..

[58] World Health Organization (1948). Summary Reports on Proceedings Minutes and Final Acts of the International Health Conference Held in New York from 19 June to 22 July 1946.

[59] Holt-Lunstad J. (2024). Social connection as a critical factor for mental and physical health: Evidence, trends, challenges, and future implications. World Psychiatry.

[60] Belgrano A., Cucchiella F., Jiang D., Rotilio M. (2023). Anthropogenic modifications: Impacts and conservation strategies. Sci. Rep..

[61] Ángel-Maya A. (2015). La Fragilidad Ambiental de la Cultura. Historia y Medio Ambiente.

[62] Mahtta R., Fragkias M., Güneralp B., Mahendra A., Reba M., Wentz E., Seto K. (2022). Urban land expansion: The role of population and economic growth for 300+ cities. Npj. Urban Sustain..

[63] Masson-Delmotte V., Zhai P., Pirani A., Connors S.L., Péan C., Berger S., Caud N., Chen Y., Goldfarb L., Gomis M.I., Intergovernmental Panel on Climate Change (2021). Summary for policy makers. Climate Change 2021: The Physical Science Basis.

[64] Medina E. (2019). La contaminación del aire, un problema de todos. Rev. De La Fac. De Med..

[65] Liu A.Y., Trtanj J.M., Lipp E.K., Balbus J.M. (2021). Toward an Integrated System of Climate Change and Human Health Indicators: A Conceptual Framework. Clim. Change.

[66] Balbus J., Crimmins A., Gamble J.L., Easterling D.R., Kunkel K.E., Saha S., Sarofim M.C.C. (2016). 1: Introduction: Climate Change and Human Health. The Impacts of Climate Change on Human Health in the United States: A Scientific Assessment.

[67] Ebi K.L., Semenza J.C. (2008). Community-based adaptation to the health impacts of climate change. Am. J. Prev. Med..

[68] Rocque R.J., Beaudoin C., Ndjaboue R., Cameron L., Poirier-Bergeron L., Poulin-Rheault R.-A., Fallon C., Tricco A.C., Witteman H.O. (2021). Health effects of climate change: An overview of systematic reviews. BMJ Open.

[69] Monroe M.C., Plate R.R., Oxarart A., Bowers A.W., Chaves W.A. (2019). Identifying Effective Climate Change Education Strategies: A Systematic Review of the Research. Environ. Educ. Res..

[70] Chang C., Pascua L. (2017). The state of climate change education—Reflections from a selection of studies around the world. Int. Res. Geogr. Environ. Educ..

[71] Caride J.A., Meira P.Á. (2019). Educación, ética y cambio climático. Innov. Educ..

[72] Sauvé L. (2017). Educación Ambiental y Ecociudadanía: Un proyecto ontogénico y político. Rev. Eletrônica Do Mestr. Em Educ. Ambient..

[73] Leff E. (2014). La apuesta por la vida. Imaginación Sociológica e Imaginarios Sociales en los Territorios Ambientales del Sur.

[74] Valderrama-Hernández R., Alcántara L., Limón D. (2017). The Complexity Of Environmental Education: Teaching Ideas and Strategies From Teachers. Procedia—Soc. Behav. Sci..

[75] Álvarez-García O., García-Escudero L.A., Salvà-Mut F., Calvo-Sastre A. (2019). Variables influencing pre-service teacher training in education for sustainable development: A case study of two Spanish universities. Sustainability.

[76] Summers J.K., Smith L.M., Case J.L., Linthurst R.A. (2012). A review of the elements of human well-being with an emphasis on the contribution of ecosystem services. Ambio.

[77] Evans T.L. (2019). Competencies and Pedagogies for Sustainability Education: A Roadmap for Sustainability Studies Program Development in Colleges and Universities. Sustainability.

[78] Amérigo M., Aragonés J.I., De Frutos B., Sevillano V., Cortés B. (2007). Underlying dimensions of ecocentric and antropocentric environmental beliefs. Span. J. Os Psycol..

